# Nanoscale diffractive probing of strain dynamics in ultrafast transmission electron microscopy

**DOI:** 10.1063/1.5009822

**Published:** 2018-01-25

**Authors:** Armin Feist, Nara Rubiano da Silva, Wenxi Liang, Claus Ropers, Sascha Schäfer

**Affiliations:** 1 4th Physical Institute - Solids and Nanostructures, University of Göttingen, Göttingen, Germany; 2 Wuhan National Laboratory for Optoelectronics, Huazhong University of Science and Technology, Wuhan, China; 3 International Center for Advanced Studies of Energy Conversion (ICASEC), University of Göttingen, Göttingen, Germany

## Abstract

The control of optically driven high-frequency strain waves in nanostructured systems is an essential ingredient for the further development of nanophononics. However, broadly applicable experimental means to quantitatively map such structural distortion on their intrinsic ultrafast time and nanometer length scales are still lacking. Here, we introduce ultrafast convergent beam electron diffraction with a nanoscale probe beam for the quantitative retrieval of the time-dependent local deformation gradient tensor. We demonstrate its capabilities by investigating the ultrafast acoustic deformations close to the edge of a single-crystalline graphite membrane. Tracking the structural distortion with a 28-nm/700-fs spatio-temporal resolution, we observe an acoustic membrane breathing mode with spatially modulated amplitude, governed by the optical near field structure at the membrane edge. Furthermore, an in-plane polarized acoustic shock wave is launched at the membrane edge, which triggers secondary acoustic shear waves with a pronounced spatio-temporal dependency. The experimental findings are compared to numerical acoustic wave simulations in the continuous medium limit, highlighting the importance of microscopic dissipation mechanisms and ballistic transport channels.

## I. INTRODUCTION

Controlling confined phononic modes in the giga- to terahertz frequency range offers new approaches to steer the flow of heat in nanoscale structures^1^ with a broad field of potential applications, ranging from advanced thermoelectric devices^2^ to the heat management in dense semiconductor circuits.^3^ Furthermore, coupled to tailored light fields, phononic modes with mega- to gigahertz resonance frequencies already developed into essential building blocks in nanometrology.^4,5^

Nanophononics based on tailored multilayer structures has made great progress in recent years, achieving, for example, phonon filtering^6^ and phonon amplification.^7^ Beyond layered systems, three-dimensionally nanostructured materials facilitate thermally rectifying behavior,^8^ highly efficient channeled thermal transport across nanoscale vacuum gaps,^9–11^ enhanced light matter interactions in combined phononic-photonic resonators,^12^ and phonon lasing.^13,14^ Optical methodologies, such as ultrafast optical spectroscopy^15^ and Brillouin scattering,^16–18^ allowed for experimental access to the spectral and temporal properties of nanophononic systems, including resonance frequencies, dissipation times,^19^ and nonlinear couplings.^20^ However, extracting quantitative information on the structural distortion in nanophononic structures often requires elaborate theoretical modeling. Knowledge of the strain field is essential for tailoring the interaction between phononic fields and other degrees of freedom, such as the coupling of lattice distortions to the electronic^21^ and magnetic^22,23^ subsystems, interaction with confined light fields,^12^ and phase-transitions driven by acoustic^24^ and optical^25^ phonon fields.

In laterally homogenous samples, ultrafast electron^26–34^ and X-ray^35–39^ diffraction allows for quantitative access to collective transient lattice distortions. Extending these approaches to three-dimensionally nanostructured geometries remains challenging, despite recent progress in ultrafast coherent diffractive dark-field imaging^40,41^ utilizing intense X-ray pulses at free-electron laser facilities.^42^ In a table-top approach, ultrafast transmission electron microscopy (UTEM)^43–51^ provides a visualization of nanophononic modes by time-resolved bright-field imaging,^52–55^ with first steps towards local diffractive probing.^56–58^ However, the full capabilities of conventional transmission electron microscopy^59–65^ for the quantitative mapping of strain fields have not been harnessed in UTEM.

Here, we demonstrate the quantitative nanoscale probing of optically triggered ultrafast strain dynamics in UTEM, employing ultrashort electron pulses in convergent beam electron diffraction (CBED). We achieve a quantitative three-dimensional spatio-temporal reconstruction of the ultrafast lattice distortions in nanoscopic volumes close to the edge of a single crystalline graphite membrane. High-amplitude coherent expansional and shear acoustic waves are launched at the symmetry-breaking sample boundaries, and we track their ballistic propagation and dephasing on nanometer length scales.

## II. ULTRAFAST CONVERGENT BEAM ELECTRON DIFFRACTION

**FIG. 1.**
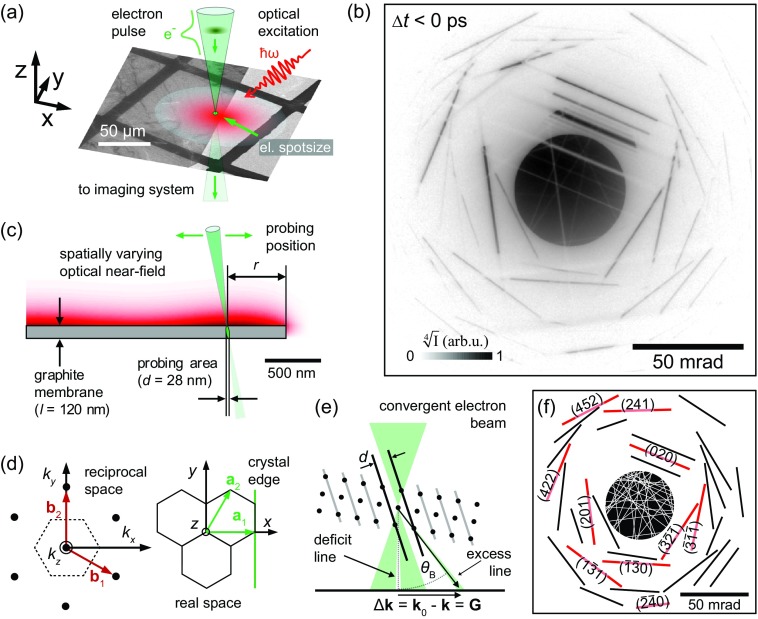
Ultrafast convergent beam electron diffraction on single crystalline graphite. (a) Local diffractive probing of optically induced (50-μm laser focal spot size) inhomogeneous structural dynamics in a single crystalline graphite membrane (background image: overview bright field electron micrograph). (b) CBED pattern before optical excitation (exemplary probing position: 500-nm distance to crystal edge). For better visibility of diffraction lines at high scattering angles, the fourth root of the electron intensity *I* is shown. (c) Experimental geometry of nanoscale probing at the graphite edge. A sharply focused electron beam (28-nm electron focal spot size) maps the local structural dynamics at a variable distance *r* relative to the edge. Optical interference leads to a slight variation of the excitation profile (sketched in the background). (d) Orientation of in-plane graphite unit cell in real and momentum space with the corresponding coordinate system (orientation of the crystal edge is indicated). (e) Bragg scattering from lattice planes (*hkl*) results in electron momentum change Δ**k **=** G**_*hkl*_, forming deficit and excess lines in the diffraction pattern. (f) Calculated deficit (white) and excess (black, red) Bragg line positions for the employed sample orientation. For selected Bragg lines, the corresponding Miller indices are given.

In the experiments, we generate low-emittance ultrashort electron pulses by localized photoemission from a tip-shaped field emitter.^46,51,66^ The femtosecond electron pulses are accelerated to an electron energy of 120 keV, coupled into the electron optics of a transmission electron microscope and tightly focused (28-nm focal spot size) onto a 120-nm thick graphite membrane. For varying probing positions relative to the edge of the membrane, electron diffraction patterns are detected in the far-field [Fig. 1(a)]. The sample is optically excited by femtosecond laser pulses focused to a 50-μm focal spot diameter (centered at the graphite edge, 800-nm central wavelength, 50-fs pulse duration, 16-mJ/cm^2^ fluence). Inhomogeneous structural dynamics are induced on length scales much smaller than the optical focal spot size due to the broken translation symmetry at the nearby sample edge. At an adjustable delay time Δ*t* relative to the electron pulse arrival, local structural dynamics are stroboscopically mapped at the electron focal spot position. See supplementary material for further details on the experimental setup and the graphite sample system.

Figure 1(b) displays a typical ultrafast large-angle convergent beam electron diffraction pattern recorded with femtosecond electron pulses before optical excitation (Δ*t *< 0). In the pattern, the central intense disc-like feature represents the angular distribution of the illuminating electron pulses. Bragg scattering conditions for the graphite lattice planes (*hkl*) are fulfilled along specific lines in momentum space.^67^ At their intersection with the central disc, efficient scattering occurs, forming deficit intensity lines within the disc, and excess lines, which are radially displaced by Bragg angles *θ*_B_ [Figs. 1(e) and 1(f)].

The angular displacement of each line encodes the length and orientation of a specific reciprocal lattice vector **G**_*hkl*_ and the scattering efficiency encodes the corrugation of the scattering potential.^62,68^ Thereby, U-CBED gives access to the ultrafast temporal change of local lattice periodicities *d_hkl_* and atomic mean-square displacements $\sqrt{\left\langle u^{2}\right\rangle }$ (supplementary material, SI 4). The broad angular range of the incident electron beam (50 mrad full convergence angle) and the chosen sample orientation enable the simultaneous observation of multiple independent Bragg scattering conditions and the corresponding rocking curves,^69^ providing direct experimental access to the local structural distortion and its temporal evolution.

After optical excitation, we observe pronounced delay-dependent radial Bragg line shifts Δ*θ* (by up to 6 mrad) in the CBED pattern. For a series of delay-dependent diffraction patterns, see supplementary material movies, M1 and M2. The induced strain dynamics results in no significant azimuthal rotation of Bragg lines for the chosen sample orientation. In the following, we therefore consider the transient changes of Bragg line profiles, obtained by integrating the diffracted intensity along the individual line directions.

**FIG. 2.**
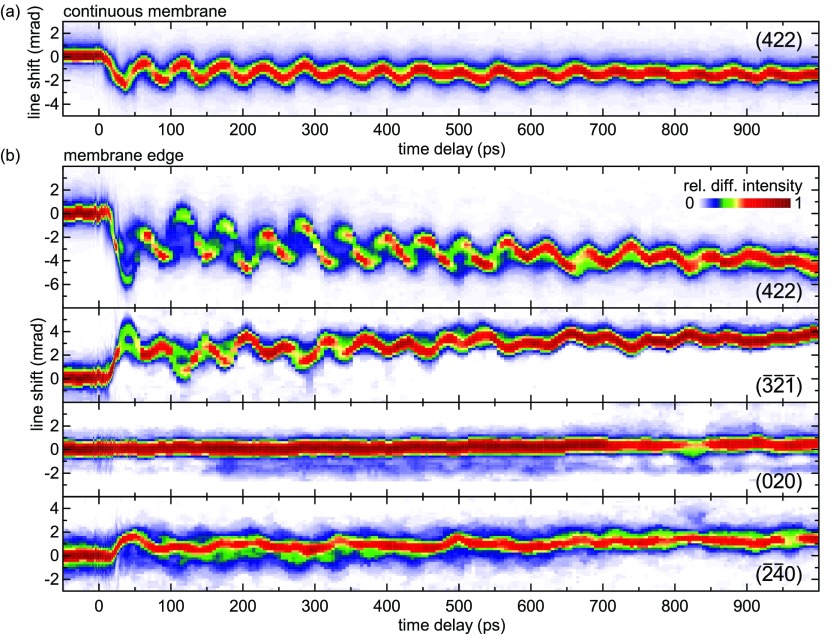
Transient modulation of Bragg line profiles. Delay-dependent profiles of selected Bragg lines for probing (a) within a continuous part of the membrane and (b) close to the graphite membrane edge (500-nm relative distance).

In Fig. 2, we show the delay-dependent profiles of selected excess Bragg lines for two different probing positions. With the electron focal spot placed at a distance of *r* = 500 nm from the edge of the graphite membrane [Fig. 2(b)], the (422) and ($\bar{3}\bar{2}\bar{1}$) lines display a strong multi-frequency oscillatory behavior of the average line position and a modulation of the line profile, even including line splittings into multiple components. Other Bragg lines show a different temporal characteristic [e.g., ($\bar{2}\bar{4}0$)] or only very weak overall changes [e.g., (020)]. Remarkably, the recorded transient changes are strongly influenced by the nearby membrane edge. In a continuous part of the film, a much simpler dynamic behavior of the line profiles is observed, as is evident by comparing the transient (422) profiles in Figs. 2(a) and 2(b).

## III. EXTRACTING THE DEFORMATION GRADIENT TENSOR

Disentangling the complex structural dynamics encoded in the ultrafast Bragg line shifts requires a quantitative description of the CBED pattern and its dependence on the distortion of the graphite film. Microscopically, the evolution of the local structural deformation of the membrane is described by the time-dependent tensor field of the deformation gradient **F**(*r*, Δ*t*)= **ε **+ **ω **+ **Ι**_3_, which can be decomposed into a symmetric strain tensor **ε** and an antisymmetric rotation tensor **ω** (**I**_3_: unit tensor).^62^

Calculating the position of deficit and excess Bragg lines in the CBED pattern requires an adequate description of the Bragg scattering conditions in reciprocal space, which we derive from the graphite unit cell^70^ defined by $a_{1}=a{\left[1,\;0,\;0\right]}^{T},\;a_{2}=a\;{\left[1/2,\;\sqrt{3}/2,\;0\right]}^{T},\;a_{3}=c{\left[0,\;0,\;1\right]}^{T}$, with lattice constants a=2.46  Å and c=6.71  Å. To account for an arbitrary sample orientation, the real space basis, represented by the matrix $B_{u}=\left[a_{1}\;a_{2}\;a_{3}\right]$ in the three-dimensional coordinate system (*x*, *y*, and *z*), is rotated [see Fig. 1(a)] by applying a matrix $R=R_{Z}(\gamma )R_{Y}(\beta )R_{X}(\alpha )$, with rotation matrices $R_{X,Y,Z}$ around a laboratory-fixed coordinate system, *X*, *Y*, and *Z* axes, respectively. Taking the planar sample orientation into account, the angles α and β correspond to the angular degrees-of-freedom of the double-tilt sample holder, and γ is related to the azimuthal orientation of the graphite flake. The reciprocal basis in the laboratory-fixed coordinate system is given by $G_{u}={\left(RB_{u}\right)}^{-1}$, so that the reciprocal lattice vector with Miller indices *h*, *k*, and *l* is expressed as $G=G_{u}{\left[h,\;k,\;l\right]}^{T}$. For scattered and incident wave vectors **k** and **k**_0_, allowed scattering conditions are obtained from $G^{2}+2k_{0}\cdot G=0$ by considering the Laue equation $G=\Delta k=k-k_{0}$ (conservation of momentum) and elastic scattering ${\left(G+k_{0}\right)}^{2}=k^{2}$ (conservation of energy).^69^ In the paraxial approximation, i.e., $k_{0X},\;k_{0Y}⪡k_{0}$ and $k_{X},\;k_{Y}⪡k$ (for the optical axis chosen along *Z*), the scattering conditions can be simplified to

$$-G^{2}/2=k_{X}G_{X}+k_{Y}G_{Y}+k_{0}G_{Z},$$ (1)

which describes straight lines $\left[k_{X},\;k_{Y}\right]$ in transverse k-space, for each reciprocal lattice vector **G**. In CBED, the incidence electron spot covers a circular region in the diffraction pattern, and, with the detector plane perpendicular to the optical axis, the allowed scattering conditions are visible as deficit lines with a distance to the origin of $r_{\text{deficit}}=\left(G^{2}/2-k_{0}G_{Z}\right)/\sqrt{G_{X}^{2}+G_{Y}^{2}}$ and an inclination angle of $\text{tan}\;(\varphi )=G_{X}/G_{Y}$.

The electrons are scattered into excess lines, which are displaced from the corresponding deficit line by the projected radial scattering vectors $\left[G_{X},\;G_{Y}\right]$, so that their radial distance becomes

$$r_{\text{excess}}=r_{\text{deficit}}+\sqrt{G_{X}^{2}+G_{Y}^{2}}.$$ (2)

Bragg line shifts are evaluated by considering changes in their center-of-mass. Additional broadening in the Bragg line profiles due to the inhomogeneous strain distributions is analyzed in Sec. VI. For the current sample system, diffusively large-angle scattered electrons only give a minor contribution to the diffraction intensity, so that only a few Kikuchi lines are visible [e.g., the deficit (010) Kikuchi line].

Considering rotation angles [α, β, γ]= [1.46°,  8.05°,  22.9°] of the graphite crystal and an initial convergence angle of 25 mrad (half angle), the precise position (radius and inclination) of deficit and excess Bragg lines in the diffractograms are reproduced, allowing for an assignment of the indices *h*, *k*, and *l* [cf. Fig. 1(f)] and further validating the use of the paraxial approximation (all utilized scattering angles are smaller than 80 mrad from the electron optical axis).

**FIG. 3.**
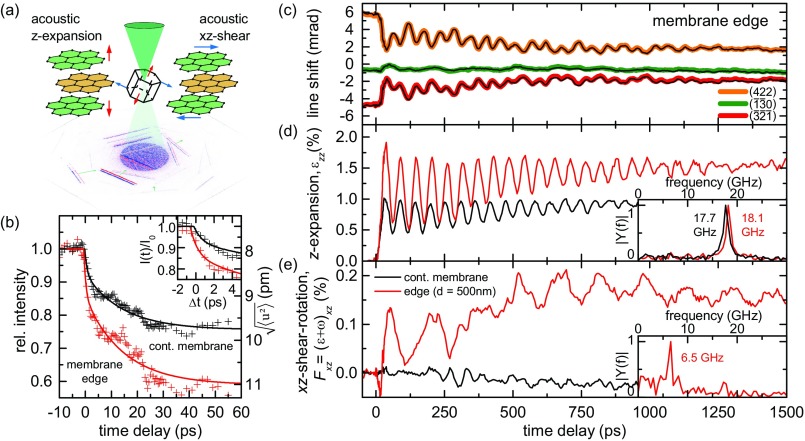
Time-dependent Bragg-line changes and dynamics of selected components of the deformation gradient tensor. (a) Local probing of the mean unit cell deformations reveals two dominating mechanical modes: an out-of-plane *z*-axis expansion and an acoustic shear-rotation in the *xz*-plane. (b) Change in (452) Bragg-line intensity and square root of atomic mean square displacement $\sqrt{\left\langle u^{2}\right\rangle }$ in the in-plane direction after optical excitation for probing at the graphite edge (red) and in a continuous part of the membrane (black). (c) Experimentally obtained delay-dependent center-of-mass shift (black line) and reconstructed mean line position (colored line, background) of the (422), ($\bar{1}\bar{3}0$), and ($\bar{3}\bar{2}\bar{1}$) Bragg lines, probed at the graphite edge. (d) and (e) Reconstructed *z*-axis expansion (d) and in-plane *xz*-shear-rotation (e) components (red: membrane edge, black: continuous membrane) with respective Fourier analysis (inset, |Y(f)|: Fourier amplitude).

**FIG. 4.**
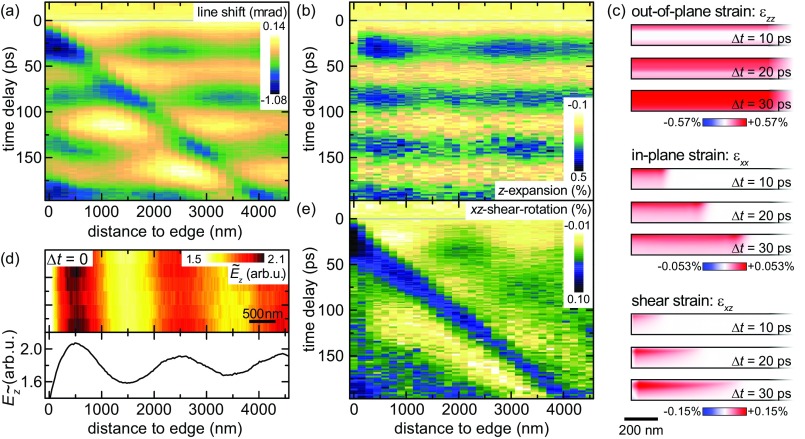
Spatio-temporal mapping of near-edge strain dynamics in single crystalline graphite. (a) Mean shift of the (201) Bragg-line as a function of time delay and probing position (recorded at a reduced optical fluence compared to the non-spatially resolved CBED data). Extracted *z*-axis expansion (b) and in-plane shear-rotation (e) retrieved by evaluating the shift of several Bragg lines. (c) Numerical finite-element simulation of the ε_*zz*_(*r*, Δ*t*), ε_*xx*_(*r*, Δ*t*), and ε_*xz*_(*r*, Δ*t*) strain tensor components (absorbed energy density adopted to match experimental ε_*zz*_ strain amplitude), illustrating the out-of-plane expansion and the in-plane propagating shock wave within 30 ps after optical excitation. (d) Characterization of the optical near-field structure by scanning photon-induced near-field electron microscopy (S-PINEM), with an optical incidence angle of about 39°.

A time-dependent distortion of the unit cell can be described by applying the deformation gradient tensor F(Δt) to the undistorted real-space basis of the graphite lattice $B_{t}(\Delta t)=F(\Delta t)\cdot B_{u}$. Extracting lattice deformations from CBED patterns is a well-established procedure in electron microscopy with continuous beams,^62,71^ which we now apply to time-resolved diffraction data. Generally, the average unit cell deformation within the electron beam probing volume (cf. Figs. 3 and 4) can be extracted by applying a forward least squares regression analysis,^72,73^ fitting the absolute change in Bragg line positions $\Delta r_{\left(hkl\right)}=r_{\text{excess},\left(hkl\right),\;\text{exp}\;}-r_{\text{excess},\left(hkl\right),\text{calc}}(F)$ and inclination angles $\Delta \varphi_{\left(hkl\right)}=\varphi_{\left(hkl\right),\;\text{exp}\;}-\varphi_{\left(hkl\right),\text{calc}}(F)$ of the most intense lines with the components of the tensor **F** as free parameters

$$\sum_{h,k,l}{\left(\Delta r_{\left(hkl\right)}(F)\right)}^{2}+\sum_{h,k,l}{\left(\Delta \varphi_{\left(hkl\right)}(F)\right)}^{2}\to \min.$$ (3)

No change of the inclination angles $\varphi_{\left(hkl\right),\;\text{exp}\;}$ is observed, and we therefore adopt $\Delta \varphi_{\left(hkl\right)}=0$ in the fitting procedure.

In our experiment, the radial position of the excess Bragg lines exhibits a high sensitivity to changes of the displacement field **u** along the *Z* direction, mainly related to the components *F_xz_* = (*ε*+*ω*)_*xz*_, *F_yz_* = (*ε*+*ω*)_*yz*_, and *F_zz_* = 1 + ε_*zz*_ of the deformation gradient tensor.^74^ Adapting these tensor components, we can quantitatively reproduce the center-of-mass shift of the selected excess lines [cf. reconstructed line positions in Fig. 3(c)]. We note that a pure membrane shear along the *x*-axis is described by a finite value of *F_xz_* and *F_zx_* = 0, so that *F_xz_*/2 = *ε_xz_* = *ω_xz_*. The full tensor **F** can in principle be determined by additionally analyzing deficit Bragg lines^71,73^ and by recording diffraction patterns along different crystal directions.^74^

Generally, in electron diffraction, rocking curves can be strongly affected by multiple scattering processes. However, for the deformation gradient tensor analysis, we only evaluated Bragg lines with extinction lengths ξ > 420 nm, which is significantly larger than the membrane thickness, so that the influence of multiple scattering effects can be neglected and kinematic scattering theory yields a good approximation.

## IV. LOCAL ULTRAFAST STRUCTURAL DYNAMICS

A quantitative analysis of the Bragg line shifts in Fig. 2 allows us to identify the complex superposition of the acoustic lattice distortions involved in the optically driven dynamics at a homogeneous part of the membrane and at its edge. The local distortion alters the spacing and orientation of crystal lattice planes, resulting in characteristic shifts of Bragg conditions in momentum space [Fig. 3(a)]. We extract the components of the local deformation gradient tensor **F** for each delay time considering the center-of-mass of multiple experimental Bragg line positions [selected traces shown in Fig. 3(c), see supplementary material, SI 3).

The temporal evolution of the tensor **F** near the membrane edge is dominated by two components *F_zz_*(Δ*t*) = 1* *+ *ε_zz_* and *F_xz_*(Δ*t*) = (*ε+ω*)_*xz*_ [Figs. 3(d) and 3(e), red curves], corresponding to an expansional strain along the graphite out-of-plane *z*-axis [for the coordinate system, see Figs. 1(a) and 1(d)] and a shear-rotation in the *xz*-plane (perpendicular to the membrane edge), respectively. Both deformations leave the (0k0) lattice planes unchanged, consistent with the experimentally found negligible transient changes of the (020) line profiles [cf. Fig. 2(b)]. Remarkably, the deformation gradient tensor analysis disentangles the multi-frequency temporal behavior of individual Bragg line shifts. The components *ε_zz_* and (*ε*+*ω*)_*xz*_ each exhibit damped oscillations at a single frequency, with periods of *T*_expansion_ = 56.5 ± 1.6 ps and *T*_shear-rot_ = 154 ± 5 ps [central frequencies of 17.7 ± 0.5 and 6.5 ± 0.2 GHz, see Figs. 3(d) and 3(e)] for the expansional and shear motion, respectively. Far from the membrane edge (150-μm distance), the optically induced deformation [Figs. 3(d) and 3(e), black curves] is primarily governed by the expansional out-of-plane motion, and no significant amplitude in the *xz*-component of **F** is observed.^75^

The periods of the expansional and shear-rotational distortion, *T*_expansion_ and *T*_shear-rot_, are given by the roundtrip time of the acoustic waves propagating between the two faces of the membrane. The ratio *T*_shear-rot_/*T*_expansion_ = 2.73 ± 0.16 is in excellent agreement with the relative magnitude of the corresponding longitudinal and transverse acoustic sound velocities in single crystalline graphite *v*(LA[001])/*v*(TA[001]) = (4140 m/s)/(1480 m/s) = 2.80.^76^ Furthermore, the periods *T* = 2 *l*/*v* yield a membrane thickness of *l* = 117 nm, which matches the value of 120 nm derived by evaluating the thickness fringes^67,77^ of the (010) Bragg line.

At long delay times (Δ*t *> 800 ps), the oscillatory membrane expansion becomes strongly damped, approaching an average graphite interlayer distance increase of about 1.5% at the membrane edge (continuous membrane: 0.9%). In order to compare these strain values to a thermal expansion model, we extract the local temperatures from the integrated diffracted intensity change of the (452) Bragg-line after optical excitation [Fig. 3(b)]. For an equilibrated phonon distribution (Δ*t* > 100 ps), a thermal Debye-Waller behavior is reached and we extract an optically induced temperature rise of Δ*T*_cont_ = 270 K at a continuous part of the membrane and Δ*T*_edge_ = 480 K at the membrane edge, which corresponds to thermal film expansions of 0.93% and 1.65%, respectively (see supplementary material, SI 4). Importantly, ultrafast CBED directly yields full transient rocking curves, so that an acoustic lattice distortion (line shift) and a change in the atomic mean square displacement (line intensity) can be distinguished.

At early delay times, a biexponential drop of diffracted intensity is observed, which is attributed to the previously reported initial non-thermal phonon distribution after optical excitation.^78–80^ This delayed increase in atomic mean square displacement is also reflected in a phase shift of the out-of-plane breathing oscillation. Specifically, we observe the first maximum of *ε_zz_* at 36 ps, corresponding to a considerable time lag of about 7 ps relative to a cosine-like transient. The quantitative relation between the non-equilibrium atomic mean square displacement and the resulting stress in the in-plane and out-of-plane directions requires further study, potentially contributing to elucidate the complex hierarchy of energy dissipation in graphite.^30,34,78–84^

The out-of-plane expansional breathing modes, visible in *ε_zz_*, are universal features observed in laser excited thin films as a result of a transient stress gradient *σ*(*z*) in the depth of the film, with electronic and lattice contributions.^15,30,34,85–89^ For the generation of shear modes, as mapped in *F_xz_*, a symmetry breaking in the lateral direction is required, such as in anisotropic or strained crystal lattices or by local light fields.^90–93^ In the following, we will further analyze the mechanism responsible for the coherent generation of these acoustic shear wave components.

## V. SPATIO-TEMPORAL STRAIN MAPPING

In our sample geometry, the structural symmetry is locally broken on mesoscopic length scales due to the presence of the membrane edge. Ultrafast CBED now allows for a local mapping of the evolving deformation gradient tensor field and the sources of the corresponding acoustic waves. To this end, we record time-resolved local diffraction patterns with the focused electron pulses placed at varying distances *r* from the membrane edge. Figure 4(a) exemplarily shows the angular shift of the (201) Bragg line as a function of the delay time Δ*t* and the probing position *r*, together with the extracted tensor components *ε_zz_*(*r*, Δ*t*) and *F_xz_*(*r*, Δ*t*) [Figs. 4(b) and 4(e)].

The expansional mode is observed at all probing positions with an equal phase. Its amplitude is spatially modulated and in particular at *r* = 500 nm is increased by about 70% compared to the value found at a larger distance from the graphite edge. This ratio agrees well with the larger temperature rise at this probing position, as observed by the transient Debye-Waller behavior [see Fig. 3(b)]. The locally increased sample excitation can be attributed to an interference pattern formed by the optical excitation close to the membrane edge, which is also observable in optically driven inelastic electron scattering, utilizing scanning photon-induced near-field electron microscopy (S-PINEM)^46,94–96^ [Fig. 4(d), supplementary material, SI 6].

In contrast to the film breathing mode, the shear-rotation component *F_xz_* shown in Fig. 4(e) exhibits a pronounced spatial dependence. In particular, the onset time of *F_xz_* scales linearly with the distance from the membrane edge, with a slope corresponding to a phase velocity of ∼22 km/s.

To further analyze the peculiar spatio-temporal strain dynamics, we numerically solve the elastodynamic wave equation for our sample geometry, considering a thermal stress model, a laterally homogeneous sample excitation profile, and graphite bulk properties for the elasticity tensor. The temperature field is obtained by taking into account the inhomogeneously deposited optical excitation and the graphite heat capacity.^97^ In addition, diffusional heat transport was included in the model using an anisotropic heat conductivity.^97^ For further details on the numerical simulations, see supplementary material.

For the *ε_zz_*(*r*, Δ*t*) component, we obtain an *r*-independent temporal evolution [Fig. 4(c), top], in agreement with the breathing mode of a continuous membrane. In addition, optical excitation results in an in-plane thermal stress *σ_x_* of the graphite lattice, which launches an expansional shock wave in *ε_xx_*(*r*, Δ*t*) from the membrane edge [Fig. 4(c), center], propagating perpendicular to the edge with the longitudinal in-plane sound velocity LA[100] = 22.16 km/s.^76^ Due to the optical excitation profile, the expansional in-plane shock wave is localized to the top of the membrane and thereby induces shearing of the thin film sample. The ultrafast build-up of shear strain at the top initiates the shear wave travelling back and forth between the membrane faces.^98^ This model readily explains the local excitation of the experimentally observed shear wave with its onset time scaling linear with the distance to the graphite edge.

## VI. TRANSIENT BRAGG LINE PROFILES

Up to here, Bragg line shifts in scanning U-CBED yielded a spatio-temporal map of the lateral structural distortion of the photo-excited graphite membrane. In addition, rich experimental information on the inhomogeneous strain within the depth of the membrane is contained in the profiles of the Bragg lines, which we analyze in the following. Within kinematic scattering theory, a strained crystal imprints a phase modulation onto the diffracted electron wavefront,^40,59^ resulting in a CBED profile well described by

$$I\left(\Delta \theta \cdot \left|G_{hkl}\right|\right)\propto {\left|F\left(e^{iG_{hkl}\cdot u\left(z\right)}\right)\right|}^{2}$$ (4)

in which Δ*θ* is the change in diffraction angle (relative to the Bragg angle *θ*_B_), **G**_*hkl*_ the corresponding reciprocal lattice vector, **u**(*z*) the atomic displacement field, and F the Fourier transformation along the graphite *z*-axis. The corresponding deformation gradient tensor **F** is given (for small deformations, as relevant here) by the gradient of the displacement field, i.e., **F ***= ***Ι**_3_ + ∇**u**. Notably, the line profiles depend on the projection **G**_*hkl*_·**u**(*z*) [Eq. (4)], so that the cross sections for individual Bragg conditions are sensitive to different components of the displacement vector field and thereby to the polarization of the involved phonon modes.

**FIG. 5.**
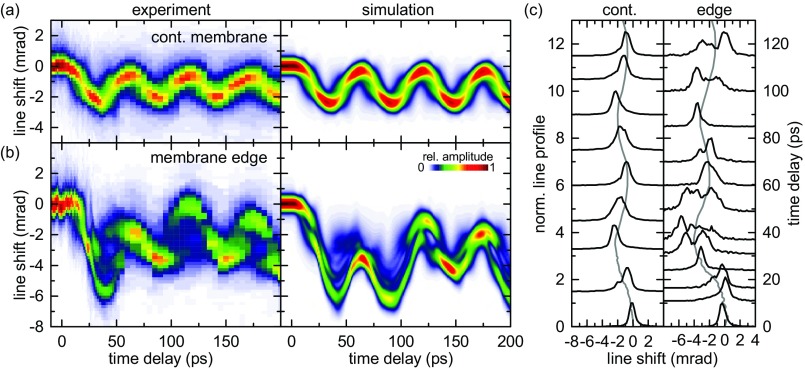
Dynamics of the (422) Bragg line profile. Extracted time-dependent cross-sections of the (422) line in (a) a continuous part of the membrane and (b) close to the graphite edge (500-nm distance) compared to calculated line profiles with displacement fields derived from numerical simulations. (c) Normalized line profiles (left-hand axis) at specific time delays (right-hand axis), with mean line position indicated in gray.

In Fig. 5, we exemplarily compare the experimental time-dependent (422) line profiles at the membrane edge and in the continuous film with predicted profiles according to Eq. (4), utilizing the numerically simulated displacement fields. For the continuous part of the graphite film, a periodic change of the Bragg line width is observed (with a period *T*_expansion_/2), which is well reproduced within the numerical strain model [Figs. 5(a) and 5(c), left panel]. Approximately at delay times of maximum film expansion and compression, sharp Bragg lines are obtained due to the intermediate nearly homogeneous *ε_zz_* strain distribution within the film, as, for example, visible in Fig. 4(c) at Δ*t* = 30 ps. The slight time lag between Bragg line shift and line broadening as well as their relative amplitude sensitively depends on the optical excitation depth and the resulting transient stress profile. In particular, the experimental width of the Bragg line profiles cannot be reproduced if one considers the optical penetration depth in graphite of *δ*_p_ = 36 nm (Ref. 99) alone. Instead, a good agreement is obtained for an excitation depth spatially spread to about 90 nm (see supplementary material, SI 5), which may be caused by fast interlayer electron or ballistic phonon transport.^100,101^ Furthermore, the asymmetry at the crests of the oscillatory Bragg line movement is reproduced well in the simulations by adopting an 8-ps coupling time of the initial excitation to the experimentally detected coherent out-of-plane motion, similar to the time constant observed for the increase of the in-plane atomic mean square displacement.^78–80^

For the strain dynamics induced at the membrane edge, the more complex behavior of Bragg line profiles [Figs. 5(b) and 5(c), right panel] is a result of the superposition of expansion and shear deformation, resulting in different projections of the displacement field **u**(*z*) onto reciprocal lattice vectors **G**_*hkl*_ [cf. Eq. (4)]. The main features of the experimental line shapes are regained in the numerical strain simulation, including the decreasing intensity maximum after Δ*t* = 0 with a pronounced line sub-structure between 23 and 60 ps [Fig. 5(b)]. In addition, also the general experimental trend of partial line re-focusing between 60 and 90 ps and increased broadening between 90 and 140 ps is found in the simulation. Microscopically, the line shapes sensitively depend on the relative amplitudes and phases of the expansional and shear wave modes, allowing for a sensitive mapping of nanophononic strain fields. The remaining difference between the experimental and simulated line profiles may indicate the break-down of classical continuum mechanics at the length and time scales considered here. Further developments are required to properly account for the impact of the initial non-thermal phonon distribution and mode specific phonon-phonon interactions on ultrafast transport processes and the transient local lattice stress, particularly relevant for the nanoscale geometries considered here.

## VII. CONCLUSION

We demonstrated the quantitative mapping of a time-dependent structural distortion in a nanoscale geometry, utilizing ultrafast convergent beam diffraction with a raster-scanned ultrashort electron probe. Our technique is applicable to a wide variety of locally structured thin-film sample systems. In particular, we believe that U-CBED opens a new avenue for achieving a quantitative description of ultrafast processes relevant in nanophononic devices, potentially allowing for a precise tailoring of nanostructure and function. With the temporal resolution demonstrated here, U-CBED is also capable of imaging phonon modes up to the terahertz regime, which will enable us to address the flow of thermal energy on its intrinsic time and length scales. Such capabilities may help to unravel the influence of local dissipation channels in complex materials, transport processes across designed interfaces, and nonlinear phononic interactions.

## VIII. SUPPLEMENTARY MATERIAL

See supplementary material for details on the experimental setup (SI 1), sample preparation (SI 2), data collection and analysis (SI 3), Debye Waller analysis (SI 4), numerical simulations (SI 5), and near field characterization (SI 6) (PDF). Movie showing delay-dependent change in CBED intensity (difference pattern) probed in a continuous part of the membrane (M 1) and close to its edge (M 2) (AVI).
